# Development of an effective clustering algorithm for older fallers

**DOI:** 10.1371/journal.pone.0277966

**Published:** 2022-11-28

**Authors:** Choon-Hian Goh, Kam Kang Wong, Maw Pin Tan, Siew-Cheok Ng, Yea Dat Chuah, Ban-Hoe Kwan

**Affiliations:** 1 Department of Mechatronics and BioMedical Engineering, Lee Kong Chian Faculty of Engineering and Science, Universiti Tunku Abdul Rahman, Kajang, Selangor, Malaysia; 2 Centre for Healthcare Science and Technology, Universiti Tunku Abdul Rahman, Kajang, Selangor, Malaysia; 3 Department of Medicine, Faculty of Medicine, Universiti Malaya, Kuala Lumpur, Malaysia; 4 Department Medical Sciences, Faculty of Healthcare and Medical Sciences, Sunway University, Bandar Sunway, Selangor, Malaysia; 5 Department of Biomedical Engineering, Faculty of Engineering, Universiti Malaya, Kuala Lumpur, Federal Territory of Kuala Lumpur, Malaysia; 6 Department of Mechanical, Materials and Manufacturing Engineering, Faculty of Engineering, University of Nottingham Malaysia, Semenyih, Selangor, Malaysia; Hefei University of Technology, CHINA

## Abstract

Falls are common and often lead to serious physical and psychological consequences for older persons. The occurrence of falls are usually attributed to the interaction between multiple risk factors. The clinical evaluation of falls risks is time-consuming as a result, hence limiting its availability. The purpose of this study was, therefore, to develop a clustering-based algorithm to determine falls risk. Data from the Malaysian Elders Longitudinal Research (MELoR), comprising 1411 subjects aged ≥55 years, were utilized. The proposed algorithm was developed through the stages of: data pre-processing, feature identification and extraction with either t-Distributed Stochastic Neighbour Embedding (t-SNE) or principal component analysis (PCA)), clustering (K-means clustering, Hierarchical clustering, and Fuzzy C-means clustering) and characteristics interpretation with statistical analysis. A total of 1279 subjects and 9 variables were selected for clustering after the data pre-possessing stage. Using feature extraction with the t-SNE and the K-means clustering algorithm, subjects were clustered into low, intermediate A, intermediate B and high fall risk groups which corresponded with fall occurrence of 13%, 19%, 21% and 31% respectively. Slower gait, poorer balance, weaker muscle strength, presence of cardiovascular disorder, poorer cognitive performance, and advancing age were the key variables identified. The proposed fall risk clustering algorithm grouped the subjects according to features. Such a tool could serve as a case identification or clinical decision support tool for clinical practice to enhance access to falls prevention efforts.

## 1. Introduction

A fall is described as an event which leads to a person unintentionally coming to rest on the ground or lower level. One in three individuals aged 65 years and above experience at least one fall over a 12 month period [1–3]. Falls are the second most common source of accidental or unintentional injury globally [4]. The cost arising from falls in the older adult comprises a sizable proportion of healthcare spending. Injuries resulting from falls include fractures and intracranial haemorrhage which subsequently lead to disability and death [5]. The negative consequences of falls adversely affect quality of life, particularly for older persons who are most likely to experience falls and injury from falls.

The mechanisms underlying falls are complex which makes the evaluation of falls and falls risk highly challenging. To deal with this, research studies have attempted fall risk estimation for older adults fall risk factors [6, 7]. Common factors considered include, but are not limited to, advanced age, muscle weakness, medications and gait imbalance. Falls are usually associated with multiple risk factors with the interaction between risk factors contributing to the risk of falls in addition to the risk associated with individual risk factors [8]. In view of the presence of numerous risk factors, the evaluation of falls risk in clinical practice is highly time consuming, requiring heavy use of the valuable but limited resource of skilled clinicians.

In recent years, dimensional reduction techniques have included feature selection and feature extraction which are applied to reduce the complexity of datasets by preserving only significant features [9]. To illustrate this, test results after various diagnoses can act as different types of features to assess the fall risk of an individual. However, the analysis may not be meaningful if irrelevant features are associated within existing data. Therefore, feature selection and extraction are essential. This can be used in isolation or combination to reduce the dimension of feature sets and improve performance for subsequent processing stages [10]. Unsupervised clustering seeks a structure in a collection of unlabelled medical data. Cluster analysis offers a standardized approach for analysing data and identifying clinically similar groups. It offers greater insights into complex medical results [11]. Learning from data can help to determine disease evolution and personalise treatments according to need [12]. Besides that, it can explore a series of underlying patterns from natural grouping which is useful for anomaly detection [13].

The purpose of this study was to develop a clustering-based fall risk algorithm that can be used as a clinical decision support system for the prevention of falls. Using clustering strength, major risk factors and characteristics for falls are discovered. This clustering algorithm is expected to group the subjects with similar features within a dataset. The clustered groups are then identified by their different fall risk. Overall, this algorithm is intended to enhance the efficiency of identification of high-risk groups for falls within the community to improve access to fall prevention initiatives.

## 2. Methods

The stages of development of the clustering algorithm included: data pre-processing, feature selection, feature extraction and clustering. These were conducted with the Spyder (Anaconda) software. In addition, the SPSS 20.0 statistical software (SPSS Inc, Chicago, IL) was used for characteristics interpretation within each clustered group.

**Fig 1:**
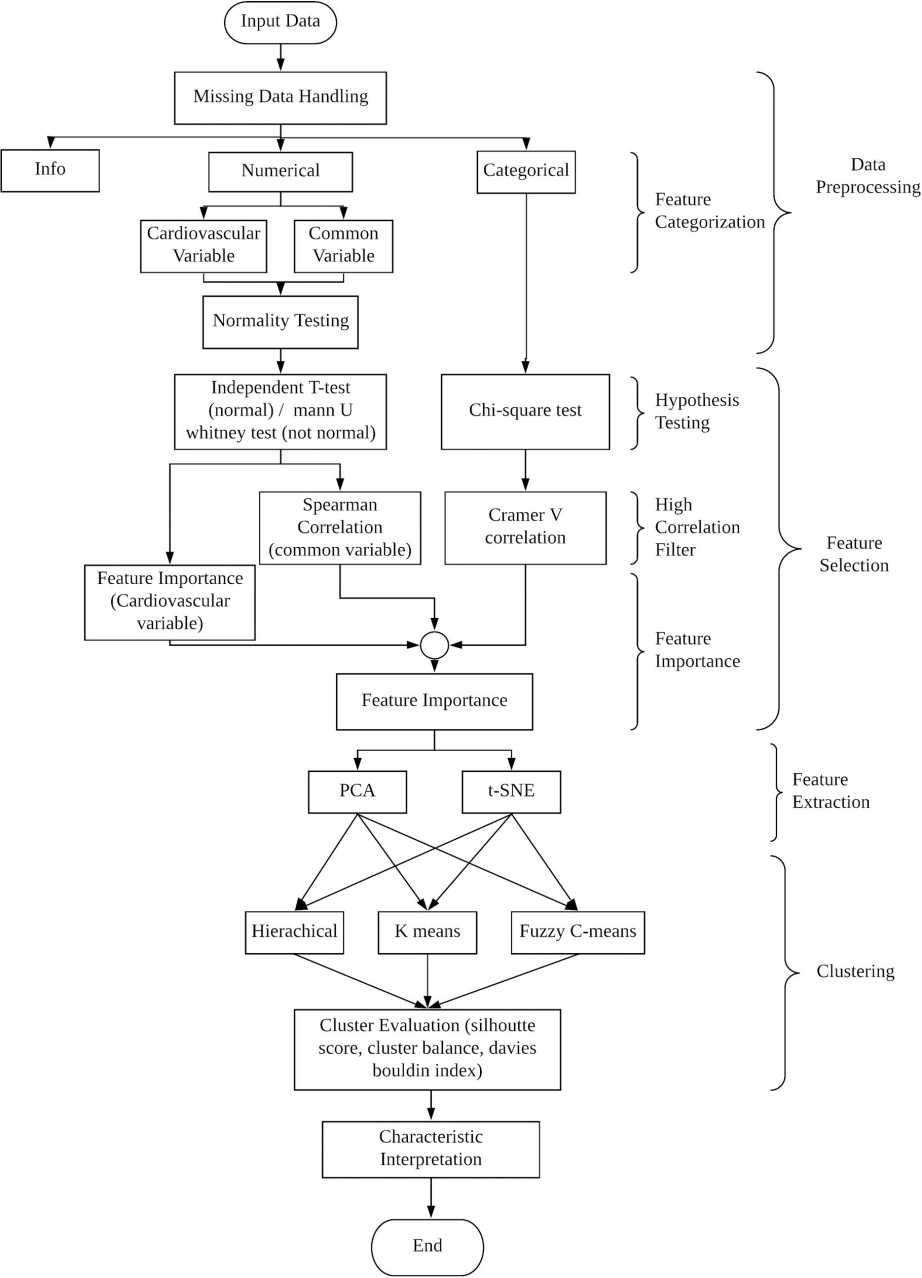
System flow diagram of the study. The input dataset was imported and analysed. In the data pre-processing stage, after missing data handing, data were categorized different categories. Numerical data were further examined with normality test and a series of feature selection steps were conducted. Categorical data skipped the normality test and proceed with feature selection stage. Then, the feature importance of the selected variables was computed. After that, feature extraction was applied, followed with different clustering algorithm. The performance was evaluated and finally the falls risk and characteristics of each clustered group were analysed.

An overview of the clustering algorithm is provided in Fig 1. At the first stage, the input dataset which obtained from the Malaysian Elders Longitudinal Research (MELoR) study was imported and analysed. In the data pre-processing stage, the algorithm remove missing data based on a predefined threshold. The data was categorized into either info (contain basic information only), categorical or numerical variables. Normality testing (Shapiro-Wilk test) was used to examine the data distribution of numerical variables. Feature selection was conducted in sequential order. Hypothesis testing (Independent T-test, Mann-Whitney U test or Chi-squared test) was then performed on each category of variables. Next, a correlation filter (Spearman correlation and Cramer V correlation) was applied on significant variables. Then, the feature importance of the selected variables was computed. After feature selection, principal component analysis (PCA) and t-distributed stochastic neighbour embedding (t-SNE) were applied to transform the chosen data into a lower dimensional space. At the clustering stage, K-means, Hierarchical and Fuzzy C-means clustering were implemented for each transformed dataset. The performance of each combination was evaluated through cluster analysis (Silhouette score, cluster balance and Davies Boulding score). The falls risk and characteristics of each clustered group were analysed by using Kruskal-Wallis H test and Dunn’s test.

### 2.1 Study dataset

The study (MELoR) dataset, was approved by the University of Malaya Medical Ethics Committee (MEC Ref No: 943.6). The aim of the MELoR study was to identify issues associated with population ageing including falls occurrence and factors associated with increased risk of falls. The MELoR study recruited community-dwelling adults aged 55 years and over, selected through simple random sampling from three parliamentary constituencies stratified by ethnicity and age deciles. The exclusion criteria of this study were: individuals who were bedbound, inability to attend the hospital healthchecks, and inability to communicate due to advanced dementia or severe speech impediments. The details of MELoR project are described in [14, 15], data on falls and cardiovascular parameters were utilized in [15]. Written informed consent was obtained from all participants at enrolment and participation was entirely voluntary, in accordance to the Declaration of Helsinki 1964, last amended 2013.

There were two stages of data collection: home-based interviews and hospital-based healthchecks. Baseline data collected were obtained through face-to-face computer-assisted interviews. The survey instrument was developed by a panel of experts in medicine, economics, law, public health, education, sports medicine, built environment, and biomedical engineering. Participants were asked a single question, whether he/she had ever experienced a fall in the preceding 12 months to determine the history of falls.

Hospital-based health checks were conducted on a separate occasion. Anthropometric measurements including standing height, weight, waist circumference and hip circumference were obtained. The Jamar Plus + digital hand dynamometer (Sammons Preston, Illinois, USA) was used to measure hand grip strength. Gait and balance were assessed using the Timed-up and Go (TUG) test, frailty walk test (15 feet walking distance) and functional reach. A detailed description on the above measures has been published elsewhere [15]. Cardiovascular autonomic reflexes were determined with the active stand [16] conducted with continuous beat-to-beat blood pressure and ECG monitoring with the Task Force ^®^ Monitor (Task Force, CNSystem, Austria) [17]. Cognitive performance was determined with the Montreal Cognitive Assessment (MoCA).

### 2.2 Data pre-processing

Data pre-processing was used to transform the raw data into understandable and accessible format. There is a total of five steps included, i.e. missing data handling, feature categorization, normality testing, feature selection, and feature extraction.

#### 2.2.1 Missing data handling

Missing data are common in the medical dataset. This study opted to exclude subjects with missing data. Percentage missing data was computed for each variable with variables exceeding 10% missing data excluded. Subjects with one or more missing variables was excluded after this step.

#### 2.2.2 Feature categorization

There are two types of data in this study. Categorical variables contain a defined set of values while numerical variables contain continuous or integer values. The univariate feature selection methods are different when dealing with categorical or numerical data. Therefore, the feature categorization step was used to classify the type of data so the feature selection method can be applied based on each type of data.

#### 2.2.3 Normality testing

The Shapiro-Wilk test was used to examine the normality of continuous variables. The null hypothesis specifies that the data is normally distributed. The variable is indicated as non-normally distributed if the test results in a p-value of less than 0.05, which rejects the null hypothesis. Variables that showed no major deviation from the normality test (accept the null hypothesis) were then assessed with histogram plots, a graphical method that is used to evaluate whether the distribution follows the familiar bell-shaped distribution. The variable was classified as normally distributed if the bell shape is observed.

#### 2.2.4 Feature selection

Feature selection algorithm was conducted to select the relevant variables from the original dataset. This was conducted through hypothesis testing, high correlation filter and feature importance in sequential order. Hypothesis testing (Independent T-test, Mann-Whitney U-test or Chi-squared test) was first performed on all variables to evaluate the difference between fallers and non-fallers. Independent T-test is commonly used to assess whether two unrelated groups are statistically different from each other, provide the data is normal distributed. A null hypothesis was stated that there is no difference between two measured variables. The probability to accept or reject hypothesis is dependent on p-value. Assume that (significance level α = 0.05), p-value obtained which less than α indicates the null hypothesis rejected, there are difference between two groups. In order to implement T-test, the variables were selected as test variables. After computation, the output significant value was used to compare with significance level, α. If it was less than significance level, such variable was significant variable and hence keep for subsequent stages. On the other hand, the variable was excluded to reduce the dimensionality as it cannot identify between faller and non-faller. The Mann-Whitney U test was performed same function as independent T-test but for the not normally distributed variable. The feature selection was conducted with Mann-Whitney U test if such variable fail Shapiro-Wilk test. As for categorical variable, Chi-squared test for independence was applied.

Next, a correlation filter (Spearman correlation and Cramer V correlation) was applied on significant variables. The Spearman correlation coefficient calculates the linear relationship between variables, with the coefficient values between -1 and +1, where 0 indicates no association, with -1 and +1 representing perfect linear association, in negative and position directions, respectively. Only one variable was chosen from highly correlated variables (correlation threshold > 0.8). Cramer V correlation was conducted for the categorical variables.

Lastly, the feature importance of the remaining variables was computed by random forest classifier. The random forest classifier is a meta estimator that applies a variety of decision tree classifiers to different sub-samples of the dataset and uses averaging to improve predictive precision and control over-fitting. In this study, it was used as a predictive model. It computes the probability of incorrect classification for the training data set decreasing with each additional variable and ranks them accordingly. The scores of each input variable in the random forest classifier indicates its relative importance for prediction. Variables with relative importance scores of < 0.05 were excluded.

#### 2.2.5 Feature extraction

Selected variables were standardized with the Z-score. Z-score normalization was used to transform the data into distribution which has a mean of zero and standard deviation of one. The variables were subsequently transformed to manageable dimensional space with Principal Component Analysis (PCA) and t-Distributed Stochastic Neighbour Embedding (t-SNE) algorithms.

The principles of PCA involve linear transformation from the input space to another dimensional space. The coordinates of data in the new space are uncorrelated and have maximal variance. It preserves only a small number of attributes [18]. The covariance matrix was obtained through *Eq 1*. The covariance matrix describes the association between the variables in the data set. It is important to recognize highly dependent variables as they contain bias and repetitive information. Besides that, covariance matrix comprising both eigenvector and eigenvalue were computed. The eigenvectors are used to classify and calculate the principal components. The eigenvalue describes the magnitude of its respective eigenvector. Computed principal components were sorted in descending order from highest to lowest eigenvalues. Only a predefined number of eigenvectors with its respective eigenvalue were chosen as they contained most of the information [19].


$$cov\;(x,y)=\frac{1}{N}\;(\sum_{j-1}^{n}\left(x_{j}-\bar{x}\right){\left(y_{j}-\bar{y}\right)}^{T})$$ Eq 1

where

N = number of samples in class

$\bar{x}$ = mean vector of input data

The t-SNE algorithm works by estimating the conditional probability of similarity points in high dimensional space. Then, t-SNE attempts to minimize the difference between similarities in the higher dimensional and lower-dimensional space [20, 21]. The Kullback-Leibler divergence was used as calculation of how the actual distribution of probability differed from the predicted distribution of probability. After this, data with minimum divergence were recreated in a lower dimensional space. The hyperparameter of perplexity was described as the effective number of neighbours for any point. Alteration of this parameter will provide different results. Therefore, repeated testing was conducted to find the most suitable perplexity for this dataset.

### 2.3 Clustering algorithm

Three clustering methods were tested in this study: K-means clustering, hierarchical clustering, and Fuzzy C-means clustering. After that, an evaluation check was conducted on the cluster, to determine whether it is well-separated from the other clusters [22]. In this case, external clustering validation was not applied because no true class label existed in this dataset. Only internal clustering validation including range, silhouette coefficient and Davies Bouldin score were computed.

K-means clustering involves two phases: 1. the centroid of each cluster was set randomly; 2. each data point was allocated to closest centroids with Euclidean distance computation according to Eq 1. The cluster centroids were continuously updated as new data points until convergence was achieved. The number of clusters, k, was determined by the elbow method (selection of the elbow in the Sum Square Error (SSE) curve) before initiating the K-means clustering.

$$d_{x,y}=\sqrt{{\left(x_{1}-y_{1}\right)}^{2}+{\left(x_{2}-y_{2}\right)}^{2}}$$ Eq 2

where

*x*_*1*_ and *x*_*2*_ = position of data point

*y*_*1*_ and *y*_*2*_ = position of centroid

Hierarchical (agglomerative) clustering with the bottom-up approach was used to form clusters. Initially, each data point was considered as one single cluster. The closest data clusters began merging into each other until the final cluster which contained all data points was formed. Dendrograms were used to illustrate the number of clusters formed with the different Euclidean distances. The number of clusters was determined by defining the minimum distance required to be a separate cluster. The criterion for choosing the pair of clusters to merge was determined using the ward method. This minimizes the total within-cluster variance.

Fuzzy C-means clustering also involved two phases: 1. the coefficients were first assigned randomly to each data point for being in the predefined clusters; 2. the centroid of each cluster was determined. The coefficient of each data point in the same cluster was computed. The cluster centroids and coefficient of each data point were continuously updated until a maximum number of iterations (1000 iterations) was achieved. The number of clusters was also decided by the elbow method before the commencement of fuzzy C-means clustering.

The range, silhouette coefficient and Davies Bouldin score were used as clustering validation methods. The range refers to the maximum cluster size difference among the clustered group. This was obtained by subtracting the number of data points between the largest and smallest clustered groups. In order to obtain the silhouette value, the mean intra cluster distance was divided by the nearest cluster distance from the next closest cluster (*Eq 3*). The value of + 1 implies the right clustering of objects, while the value of—1 means that items are not correctly clustered. Apart from that, the Davies Bouldin score was determined by the ratio between within-cluster distances and between-cluster distances (*Eq 4*). The minimum score is zero and better clustering result is indicated by a lower value.


$$S_{i}=\frac{b_{i}-a_{i}}{\max(a_{i}{,b}_{i})}$$ Eq 3

where

a = average dissimilarity inside cluster

b = average dissimilarity to neighbouring cluster


$$D_{ij}=\frac{d_{i}-d_{j}}{d_{ij}}$$ Eq 4

where

d_i_ = average distance between every data point in cluster i and its centroid

d_j_ = average distance between every data point in cluster j and its centroid

d_ij_ = Euclidean distance between the centroids of the two clusters

### 2.4 Cluster analysis

Falls risk was calculated for each clustered group by dividing the number of fallers with the total number of fallers and non-fallers within the group. Risk levels were also computed by dividing the group falls risk by the overall falls risk. Besides that, the characteristics of each group was indicated by computing the mean and standard deviation of each selected variable. The median and interquartile range was computed if the variable was not normally distributed. The data normality is important to be identified because a parametric test has more statistical power, more is likely to detect significant difference when they truly exist.

The SPSS software was then used to conduct the Kruskal-Wallis H and Dunn’s tests. The Kruskal-Wallis H test is a multiple comparison test to identify differences between groups. The null hypothesis states that there is no difference between groups. If the obtained p-value is less than 0.05, the null hypothesis is rejected. The Dunn’s test was used as a post-hoc test to perform multiple pairwise comparisons.

## 3. Results

### 3.1 Outputs of data pre-processing

The initial MELoR dataset contained 1411 subjects and 139 variables (refer S1 Table). 132 out of 139 variables had less than 10% missing data and were included in the processing step. The hypothesis testing methods successfully reduced these 132 variables to 58 variables, followed by correlation method (55 variables), and the feature importance method (9 variables). Subjects with missing data in these selected 9 variables were excluded. After data pre-processing, a total of 1279 subjects and 9 variables were selected. These selected variables comprised major fall risk factors such as gait and balance, muscle strength, cardiovascular disorder, cognitive impairment and age.

**Fig 2:**
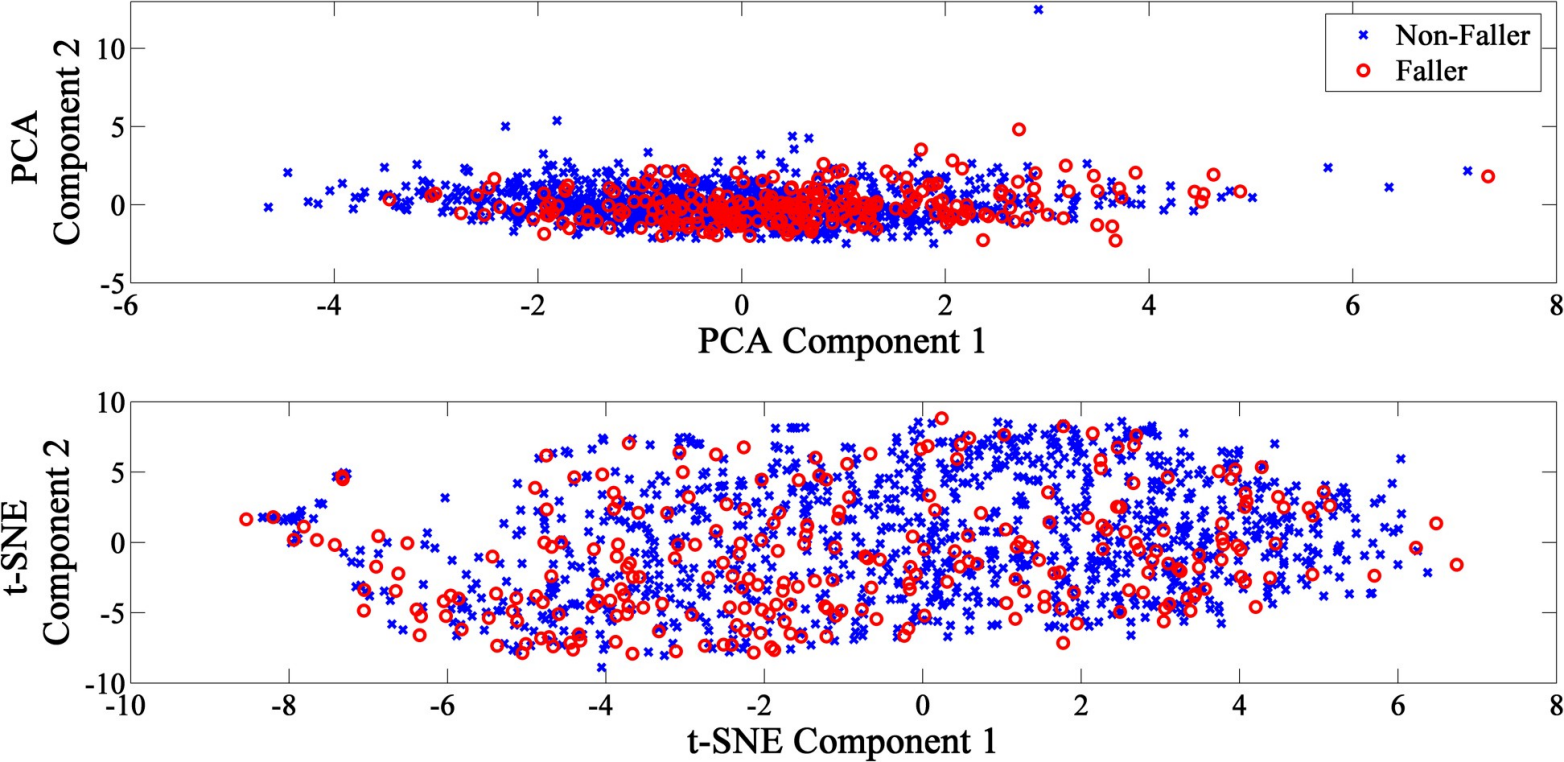
The first two components extracted from the feature extraction algorithm. (top) PCA feature extraction; (bottom) t-SNE feature extraction.

After *Z-score* normalization, the data were rescaled to compare different variables. All nine variables were computed as principal components. The first to ninth principal components represented 28.25%, 14.33%, 12.73%, 10.52%, 9.49%, 8.24%, 6.52%, 5.87% and 4.05% of the variance. In order to obtain at least 60% variance ratio, first four components (65.83% cumulative variance) were chosen. The scatter plot of data points based on first two principal components was shown in Fig 2 (top).

The t-SNE was used to create a two-dimensional representation from the original nine dimensions. By using t-SNE for feature extraction, the data were then transformed to the 2-dimensional space as shown in Fig 2 (bottom).

### 3.2 Implementation and evaluation of clustering algorithms

According to Table 1, a combination of t-SNE and k-means clustering was ranked number one among all six models. On the other hand, PCA and hierarchical clustering was the lowest performance combination because it had the most imbalanced clusters and lowest silhouette coefficient. Although a combination of PCA and Fuzzy C-means clustering had the most balanced group sizes, its performance in Silhouette Coefficient and Davies-Bouldin score were not comparable with the combination with t-SNE.

**Table 1:** The comparison of performance among different combination of dimensional reduction technique and clustering algorithm.

Combination	Cluster	Clustering Validation Methods			
		Range	Silhouette Coefficient	Davies Bouldin Score	Rank
**PCA and k-means**	5	545 ^(5)^	0.29 ^(4)^	0.88 ^(4)^	5
**PCA and Hierarchical**	4	771 ^(6)^	0.26 ^(6)^	0.96 ^(5)^	6
**PCA and Fuzzy C-means**	2	11 ^(1)^	0.27 ^(5)^	1.37 ^(6)^	4
**t-SNE and k-means**	4	149 ^(4)^	0.41 ^(1)^	0.78 ^(1)^	1
**t-SNE and Hierarchical**	4	82 ^(2)^	0.38 ^(3)^	0.85 ^(3)^	3
**t-SNE and Fuzzy C-means**	4	129 ^(3)^	0.40 ^(2)^	0.79 ^(2)^	2

Note: The rank is evaluated by least stack ranking from all three clustering validation methods.

^1, 2, 3, 4, 5, 6^ Ordinal Ranks 1 to 6

**Fig 3:**
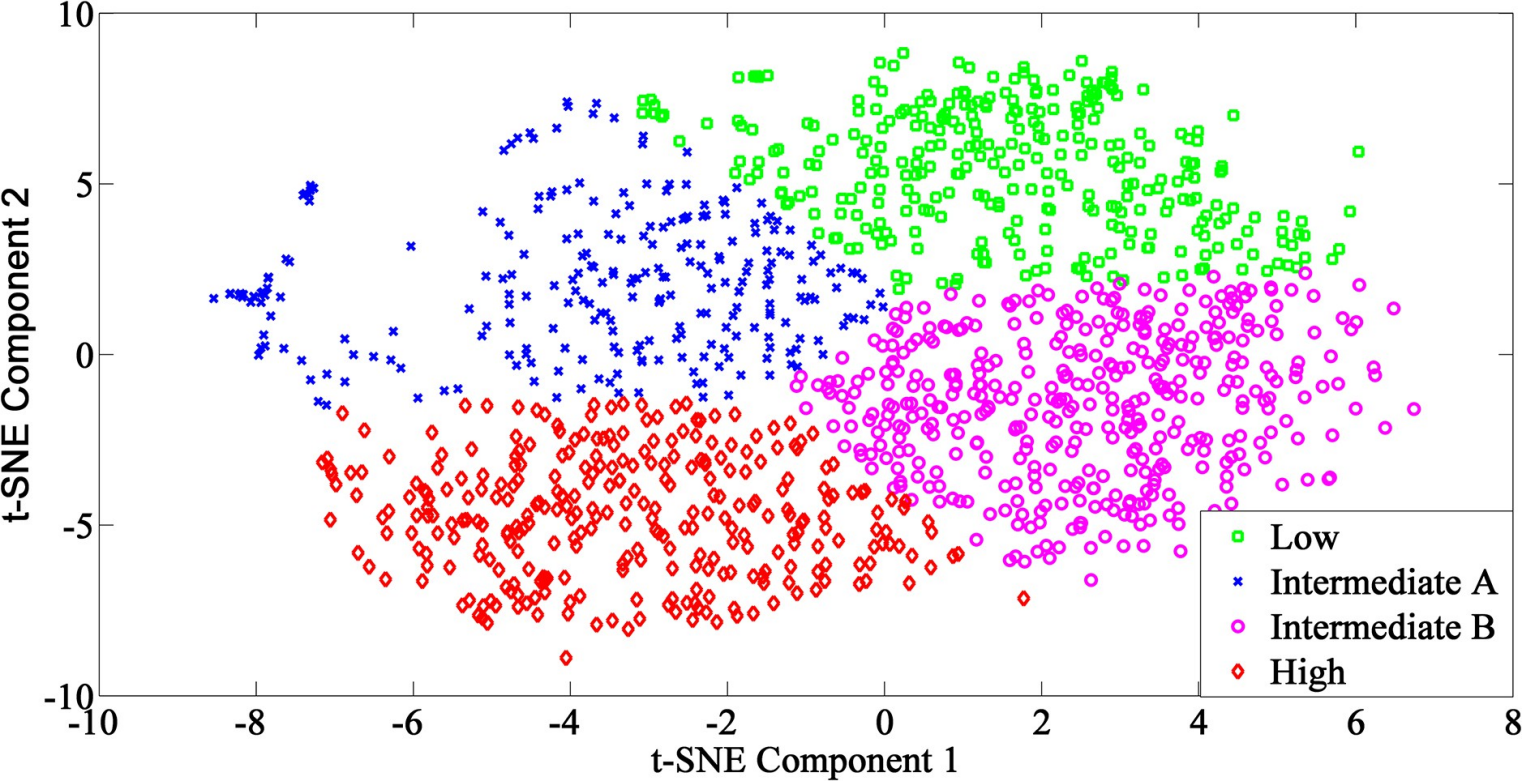
Clustering result generated by k-means clustering algorithm using features extracted from t-SNE. Low, Intermediate A, Intermediate B, and High represent the four clusters and grouped based on their similarity.

Therefore, the final chosen model was the combination of t-SNE and k-means clustering, as shown in Fig 3. Four clustered groups were identified as low, intermediate A, intermediate B and high falls risk groups. According to the trend, the falls risk was likely to increase when the data point moved downward or leftward. As illustrated in Fig 3, the low falls risk group was located at top right-hand side while the high fall risk group located at bottom left hand side. The intermediate A and B fall risk groups were located in between low and high fall risk groups.

### 3.3 Cluster characteristics

Table 2 shows the percentage of fallers classified within the low, intermediate A, intermediate B, and high falls risk groups, at 13%, 19%, 21% and 31%, respectively. 264 (21%) of the 1279 subjects eventually included in the analysis had at least one fall in the 12-month prior to the date of the interview. In comparison, the low falls risk group had an risk level of 0.62 whereas the high fall risk group had risk level of 1.48.

**Table 2:** Characteristic of clustered groups.

Variables	Fall Risk Group				p-value
	Low (n = 313)	Intermediate A (n = 406)	Intermediate B (n = 257)	High (n = 303)	
Fall Risk (%)	13	19	21	31	-
Risk Level	0.62	0.90	1.00	1.48	-
Timed Up and Go Test (TUG) (s)	11.0 ± 3.00 ^a^^,^^b^^,^^c^	11.0 ± 2.00 ^a^^,^^e^	12.0 ± 3.00 ^b^^,^^f^	15.0 ± 6.00 ^c^^,^^e^^,^^f^	***
Functional Reach Test (FR) (cm)	32.0 ± 8.00 ^b^^,^^c^	26.0 ± 7.00 ^d^^,^^e^	26.0 ± 8.00 ^b^^,^^d^^,^^f^	19.0 ± 7.00 ^c^^,^^e^^,^^f^	***
Hand Grip Strength (HGS) (kg)	32.0 ± 9.4 ^a^^,^^b^^,^^c^	19.8 ± 6.43 ^a^^,^^d^^,^^e^	24.8 ± 7.83 ^b^^,^^d^^,^^f^	17.3 ± 7.03 ^c^^,^^e^^,^^f^	***
Systolic Blood Pressure Variability Ratio	1.3 ± 0.75 ^c^	1.2 ± 0.56 ^d^^,^^e^	1.2 ± 0.68 ^d^^,^^f^	1.0 ± 0.52 ^c^^,^^e^^,^^f^	***
Diastolic Blood Pressure Variability Ratio	1.2 ± 0.60 ^a^^,^^c^	1.1 ± 0.53 ^a^^,^^d^^,^^e^	1.2 ± 0.70 ^d^^,^^f^	1.0 ± 0.45 ^c^^,^^e^^,^^f^	***
RR Variability Ratio	0.8 ± 0.41 ^a^^,^^b^^,^^c^	0.8 ± 0.48 ^a^	1.0 ± 0.99 ^b^	0.8 ± 0.51 ^c^	***
Cognitive Assessment (MoCA questionnaire)	26.0 ± 4.00 ^a^^,^^b^^,^^c^	26.0 ± 4.00 ^a^^,^^d^^,^^e^	22.0 ± 6.00 ^b^^,^^d^^,^^f^	18.0 ± 7.00 ^c^^,^^e^^,^^f^	***
Age (years)	65.7 ± 8.40 ^a^^,^^b^^,^^c^	64.2 ± 9.07 ^a^^,^^d^^,^^e^	71.6 ± 8.50 ^b^^,^^d^^,^^f^	73.1 ± 10.02 ^c^^,^^e^^,^^f^	***
Height (cm)	166.0 ± 10.00 ^a^^,^^b^^,^^c^	153.0 ± 6.00 ^a^^,^^d^	162.0 ± 8.00 ^b^^,^^d^^,^^f^	153.0 ± 10.00 ^c^^,^^f^	***

** p<0.01(conducted with Kruskal-Wallis H test)

*** p<0.001(conducted with Kruskal-Wallis H test)

Dunn’s test:

^a^ p<0.05 for Low versus Intermediate A fall risk group

^b^ p<0.05 for Low versus Intermediate B fall risk group

^c^ p<0.05 for Low versus High fall risk group

^d^ p<0.05 for Intermediate A versus Intermediate B

^e^ p<0.05 for Intermediate A versus High fall risk group

^f^ p<0.05 for Intermediate B versus High fall risk group

All selected variables were significantly different between the groups with the Kruskal-Wallis H test. Moreover, all these variables were also significantly different between low and high fall risk groups in the post-hoc analysis.

## 4. Discussion

Filter methods play an important role at the initial stage of processing to exclude irrelevant variables. In comparison, the filter methods (hypothesis testing and correlation method) are less computationally expensive than the embedded method (feature importance method) [23]. Therefore, the embedded method was applied when fewer variables remained. The result obtained from our combination was satisfactory. The final selected variables were carrying most of the information from the original set of variables. It reduced algorithm complexity and noise caused by misleading data. Thus, the relationship between these variables and falls risk was discovered.

Principal component analysis is a linear feature extraction method that aims to optimize variation and maintains large pairwise distances. However, the data set may have the manifold instead of linear structure [21]. Unlike PCA, t-SNE achieves a better visualization result in which data points are evenly spread. The data points of fallers were concentrated towards one side of the graph. This may because t-SNE is a nonlinear technique that preserve local similarities [21]. Nevertheless, in order to illustrate high-dimensional data on low-dimensional, non-linear manifolds, t-SNE with the capability to ensure similar data points could be expressed close together. In point of fact, falls incidence are known to be caused multifactorial risk factors [5], this explained the manifold structured of falls dataset.

In fact, t-SNE works with either linear or nonlinear data sets and produces meaningful clustering. Balamural and Melkumyan (2016) reported that t-SNE can retain local structure and revealed the global structure such as presence of clusters [24]. This suggests that t-SNE works with the clustering algorithm to produce a better result. Derksen, L reported that the constructed PCA before t-SNE was able to improve the result when the number of variables exceeded fifty [25]. However, this method was not implemented in this study because the number of variables has been reduced to nine only.

The combination of t-SNE and K-means clustering was ranked number one among all six combinations, as shown in Table 1. On the other hand, the combination of PCA and hierarchical clustering had the lowest performance combination because it had the most imbalanced clusters and lowest silhouette coefficient. In the evaluation with the silhouette coefficient and the Davies-Bouldin score, the PCA input data achieved lower performance compared to t-SNE input data. This indicates that the intra-cluster similarity and inter-cluster differences were lower for clusters formed by PCA input data. Therefore, t-SNE resulted in more valid and compact clusters which outperformed PCA in our study dataset. In the comparison of the clustering algorithm, Fuzzy C-means produced more compact clusters whereas K-means clustering yielded more distinct clusters [26]. In addition, K-means clustering outperforms Fuzzy C-means clustering in terms of computing time [27]. Therefore, the combination of t-SNE for feature extraction and K-means clustering algorithm achieved the best clustering performance for the MELoR dataset.

The increased odds of falls occurrence was clearly observed in the high falls risk compared to low falls risk group, with those clustered within the low falls risk group exposed to almost 40% lower odds of falls in the study population. In contrast, an almost 50% increased odds of falls was observed in the high falls risk group. This demonstrates clear discrimination of risk profile based on clustering.

The TUG completion time was significantly longer for the high falls risk group, which was recorded as 15s. A longer completion time in the TUG test (>13.5 seconds) has been found to be associated with increase in risk of falling among older persons [28]. Alexandre et al. [29] reported 12.47 seconds as the threshold value. Therefore, the falls risk of the other three groups were equivalently lower because of their TUG completion time fell below threshold value. In addition, TUG test also is the indicator of poor muscle strength, with a significant moderate correlation of r = -0.568 [30].

The functional reach (FR) decreased from the lower to the higher falls risk group. The maximal reach within 15.24 cm to 25.40 cm in FR was subjected to a doubling in falls risk [31]. From the result, the low to intermediate falls risk groups achieved a maximal reach of greater than 25.40 cm, indicating a lower falls risk. A lower score represents poorer dynamic balance hence indicating an increased risk of falls [32]. Individuals with a FR less than 18.5 cm were considered at higher falls risk [33]. This was similar with the result obtained which was 19 cm for the high falls risk group.

As for hand grip strength (HGS), the grip strength measured was lowest in the high falls risk group. HGS is known to be associated to falls [34] and it is the simplest and recommended method to assess muscle mass strength. Studies reveals that hand grip strength is correlated with fall incidence, health-related quality of life, hospitalization duration, and disability [30, 34]. Besides that, the HGS score differed according to gender [35]. Based on the result obtained, the low and intermediate B falls risk groups had a large proportion of men whereas the intermediate A and high falls risk groups had a large proportion of women. Therefore, the score was lower as expected for the intermediate A and high falls risk groups.

The high falls risk group had lower systolic and diastolic blood pressure variability ratios. Short-term blood pressure variability (BPV) provides a measurement of changes in blood pressure with posture change. Reduction in blood pressure variability demonstrate possible reduction in reactivity in blood pressure control for the upright posture [15]. This is considered as autonomic disorder and may possess a direct effect on the susceptibility to falls. However, no difference in heart rate variability was observed between low and high falls risk groups.

A lower score in the MoCA test was observed in the high falls risk group. The intermediate B and high falls risk groups had mean MoCA score below 26, indicating poorer cognitive performance. Dementia or poorer cognition is associated with a higher risk of falls [3].

In general, fall risk increases with age [3]. As reported in a systematic review, one third of those aged 65 and over, and half of those aged over 85, falling each year with a high percentage of these persons even falling several times per year [5]. Gait problems, impaired balance and weaker muscle strength are also associated with advancing age [36]. It is unsurprising, therefore, our study showed that participants aged 73.1 years had a higher falls risk compared to those aged 65.7 years.

Classification models being applied within clinical decision support system is not a new concept. A study has developed a clinical decision support system that assists clinicians and mental health practitioners in classifying and characterizing patients with depression intelligently, by analysing patient data based on their represented symptoms. In order to make relevant decisions, classification models based on online health information resources from within an electronic medical record system are used [37]. Therefore, the algorithm developed and presented here may potentially be useful as a fall risk prediction tool where the clinician is just required to enter the necessary parameters and the algorithm will automatically assign the older adult to a cluster. The odds of falling will then be estimated based on recorded fall-related parameters in the assessment. This would be helpful to identify the key fall risk factors that might contribute to falls in the older adult to inform management. In addition, this may also be used as a decision aid with regards to whether to prescribe falls risk increasing drugs for instance [38].

This study was limited to one cross-sectional dataset. The algorithm, therefore, may perform differently if applied in datasets from other studies or included as a screening instrument in clinical practice. Future work should evaluate the algorithm on other datasets or in the clinical, real-world, setting. In addition, other risk factors such as visual impairment, use of assistive device, medications and environment hazard could also be considered in future clustering analyses.

## 5. Conclusion

The proposed clustering algorithm successfully clustered the study population into four groups: low (13%), intermediate A (19%), intermediate B (21%) and high (31%) falls risk groups. Participants clustered in the high falls risk group possessed 50% higher odds of falls compared to the overall dataset. Participants included in the high falls risk group had slower gait, poorer balance, weaker muscle strength, presence of cardiovascular disorder, poorer cognitive performance and advancing age. The clustering algorithm represents a potential clinical decision support tool to identify high risk fallers for falls prevention initiatives, thus improving case finding and reducing the burden on the currently limited resource of clinicians trained in managing older adults.

## Supporting information

S1 Table Types of variables in analysis. (PDF)
