# Supplementary material for: Development of an effective clustering algorithm for older fallers
Source: PLoS One. 2022 Nov 28;17(11):e0277966. doi: 10.1371/journal.pone.0277966 (PMC9704618; doi:10.1371/journal.pone.0277966)
Supplement: S1 Table — (PDF) [file pone.0277966.s001.pdf]

Supplemental Information

Table S1: Types of Variables in Analysis

**Type of Variables**	**Number of Variables**
Informative	3
Falls questionnaire (Yes/No)	1
Demographic (age, gender, ethnicity)	3
Physical assessment (height, weight, waist and hip measurements, and etc.)	6
Muscle strengths (hand grip strength)	3
Gait (Timed-up and Go, frailty walking test)	2
Balance (functional reach)	1
Cognitive (MoCA score)	2
Cardiovascular-related in active stand test (included both blood pressure and heart rate parameters)	118
**Total**	**139**
